# Can an Evidence-Based Mental Health Intervention Indirectly Benefit Caregivers and Peers of Intervention Participants in Rural Sierra Leone?

**DOI:** 10.3390/ijerph22060844

**Published:** 2025-05-28

**Authors:** Alethea Desrosiers, Kathryn Noon, Matias Placencio-Castro, Nathan B. Hansen, Musu Moigua, Theresa S. Betancourt

**Affiliations:** 1 Department of Psychiatry and Human Behavior, Brown University, 345 Blackstone Blvd, Providence, RI 02906, USA; 2 School of Social Work, Boston College, Chestnut Hill, MA 02496, USA; noonk@bc.edu (K.N.); placenci@bc.edu (M.P.-C.); theresa.betancourt@bc.edu (T.S.B.); 3 College of Public Health, University of Georgia, Athens, GA 30602, USA; nhansen@uga.edu; 4 Caritas-Freetown, Freetown, Sierra Leone; mmoigua817@gmail.com

**Keywords:** networks, caregivers, peer group, Sierra Leone, mental health, rural

## Abstract

This study explored potential indirect mental health benefits of the Youth Readiness Intervention (YRI) among peers and caregivers of YRI participants and control participants via a networks psychometrics approach. We recruited and enrolled index participants who participated in an implementation trial in Sierra Leone (N = 165 control index participants; N = 165 YRI index participants). Index participants nominated three of their closest peers (N = 879) and one cohabitating caregiver (N = 284) to complete quantitative assessments on mental health and functioning. We used network psychometrics to explore patterns of association between mental health outcomes and risk/protective factors among YRI participants’ peers and caregivers and those of non-participants. Models of network structures showed several strong associations between mental health symptoms and risk/protective factors. There was a strong association between higher social support and positive coping skills. Additionally, models reflected stronger associations between higher depression symptoms and worse emotion regulation for peers of non-participants only. For caregivers of non-participants, a higher burden of care was strongly associated with worse emotion regulation, which was associated with higher levels of depression and anxiety. On a broader scale, the findings may provide support for wider societal benefits that evidence-based mental health interventions can offer in resource-constrained settings.

## 1. Introduction

In low- and middle-income countries (LMICs) and other low-resource settings, the burden attributable to mental health disorders is often compounded due to the widening mental health treatment gap [1,2,3]. Such dynamics are particularly pressing in post-conflict settings. For example, in Sierra Leone, a small country in West Africa that has endured numerous hardships (i.e., civil war, an Ebola Virus Disease (EVD), political instability), the mental health treatment gap is estimated at 98% [3]. While some evidence-based mental health interventions have demonstrated feasibility and effectiveness in this post-conflict setting [4,5,6,7], human and financial resource constraints can limit their reach and sustainability. Understanding whether and how evidence-based mental health interventions might offer wider societal benefits through phenomena like “spillover” or indirect effects, which are defined as beneficial effects experienced by non-participants [8], could help address health system resource constraints in Sierra Leone and similar resource-constrained settings.

One potential strategy to factor in wider societal benefits of mental health interventions is to account for how participation in mental health interventions may indirectly improve the mental health and well-being of other, non-participant household members, also referred to as “spillover effects” [9,10,11,12]. Prior studies in high-income countries have found that family members living with individuals experiencing mental health problems report significantly poorer quality of life and health status, as well as poorer mental health [13,14]. Research on indirect benefits of mental health interventions in LMICs is limited [8,12], but findings from one prior study in Sierra Leone indicated that caregivers of youth who participated in a mental health intervention experienced a reduced sense of caregiving burden and psychological distress after the intervention was finished [12].

In addition to household members, youth participation in an evidence-based mental health intervention could also indirectly benefit their peers and close friends. Research in LMICs is sparse in this area as well, but some prior studies in post-conflict settings have found that mental health and well-being are related to peer networks, particularly through mechanisms like sharing positive coping skills and bolstering social networks [15,16]. Engaging social networks in positive ways has been shown to contribute to greater self-confidence and efficacy, higher-quality relationships, and improved resilience, while reducing the time engaged with mental health and psychosocial support services [17,18,19,20]. These preliminary findings point to the potential for evidence-based mental health interventions to have larger benefits across peers and caregivers and also the need for further research to understand whether and how these benefits occur.

In prior research in Sierra Leone, we applied qualitative and mixed methods approaches to understand how participation in an evidence-based mental health intervention—the Youth Readiness Intervention (YRI; 5)—might indirectly benefit the mental health of primary caregivers and peers of at-risk youth (i.e., those with poor emotion regulation and daily functioning) who participated in the intervention [21,22]. The findings showed that YRI participants’ caregivers reported a decreased sense of caregiving burden and an improved sense of well-being, which were attributed to YRI participation and the noted improvements in behavior and functioning among their youth [21]. Similarly, close peers of YRI participants also noted improvements in their mental health and interpersonal functioning, and they perceived that these improvements were related to their indirect exposure to the YRI [21].

While these previous studies provide some insight into whether and how an evidence-based mental health intervention like the YRI may have broader mental health benefits among close peers and caregivers, little is known about how patterns of mental health symptoms and associated risk and protective factors might differ between intervention-exposed and unexposed household members and/or peers. Using novel analytic methods may help shed light on the inter-relationships between multiple mental health symptoms and potential risk and protective factors associated with mental health [23]. Network psychometrics is a relatively novel approach in psychology and psychometrics that reconceptualizes traditional views of psychological constructs [23]. Instead of treating psychological traits, symptoms, or behaviors as the result of latent variables (i.e., underlying mechanisms), network psychometrics conceptualizes and operationalizes them as networks of directly interacting components or variables [24,25]. Network psychometrics could help identify patterns of associations between mental health symptoms and factors often influencing mental health (i.e., social support, emotion regulation), which could help inform tailoring of interventions to better address “hot spots” of unaddressed mental health needs, or to bolster informal strengths and supports that could promote youth mental health. To our knowledge, this approach has not been applied to understanding how potential risk and protective factors relate to mental health and well-being across peer networks of youth and/or their household members in Sub-Saharan Africa, nor how these patterns might differ between those who have been indirectly exposed to a mental health intervention and those who have not been exposed.

As such, the current exploratory study sought to apply the networks psychometric framework to explore the associations between several mental health outcomes (i.e., depression, anxiety, well-being, quality of life) and risk and protective factors shown to relate to mental health symptoms in prior research (i.e., functional impairment, social support, emotion regulation, coping skills; [26,27,28,29]). The selection of risk and protective factors was also guided by the YRI theory of change, which highlights improved interpersonal skills and social support, emotion regulation skills, daily functioning, and positive coping as potential mechanisms linked with improved mental health [5,30]. This study’s aims were to: (a) better characterize the constellation (or patterns) of associations between mental/behavioral health symptoms and risk/protective factors for both peers and caregivers of YRI participants in Sierra Leone; and (b) explore whether and how these patterns differ between peers and caregivers of YRI participants and those of control participants. We hypothesized that (a) lower levels of protective factors would be strongly linked to higher levels of mental and behavioral health problems; and (b) peers and caregivers of YRI participants would show a qualitatively different pattern of association between mental health and risk/protective factors than those of non-participants.

## 2. Methods and Materials

### 2.1. Participants and Setting

The current study uses a cross-sectional design that includes baseline data from a larger NIMH-funded study (R01 MH113759) that was linked with a hybrid-implementation effectiveness trial of the YRI in Sierra Leone (Youth FORWARD/U19 MH109909). In this larger study, study research assistants recruited index participants (aged 18–30) who had previously enrolled in the hybrid-implementation effectiveness evaluation of the YRI implemented within entrepreneurship training. We obtained informed consent and enrolled index participants who had completed the YRI delivered within entrepreneurship training (N = 165) and control index participants (N = 165; N = 330 total). All index participants had previously provided consent to be re-contacted in relation to the current study, and provided their contact information. All youth who participated in the hybrid trial exhibited difficulties with emotion regulation and daily functioning, as measured by elevated scores on the Difficulties in Emotion Regulation Scale [31] and the World Health Organization Disability Assessment Schedule [32]. Index participants were recruited across three rural districts in the Eastern region of Sierra Leone: Koinadugu, Kono, and Kailahaun. The economy in the Eastern region is largely based on small-scale mining and agricultural production.

Index participants who enrolled in this study completed an ego network survey in which they nominated three of their closest peers (i.e., friends they shared information with and felt emotionally close to) and provided contact information for their nominated peers. We then recruited nominated peers to participate in the current study (N = 879). Inclusion criteria for peers were (a) being a part of an index participant’s peer network; and (b) aged 18 or over. Exclusion criteria were (a) being a current YRI participant or (b) exhibiting severe, active suicidality or psychosis as assessed via the MINI-SCID (Mini Structured Clinical Interview for DSM-V). Index participants also nominated one primary cohabitating caregiver (i.e., the person in their household they felt cared most for their well-being and whom they felt emotionally closest to) and provided their contact information. We then recruited nominated caregivers for participation in the current study (N = 284). Inclusion criteria for caregivers were (a) being identified as a primary adult caregiver (aged 18 or older) of a Youth FORWARD study participant; and (b) residing in the household of a Youth FORWARD participant. Exclusion criteria were (a) not residing in the household of an index participant; (b) severe, active suicidality or psychosis as assessed via the MINI-SCID; and (c) serious cognitive impairments that might inhibit one’s ability to comprehend informed consent and participate in the interview. This study was approved by the Boston College Institutional Review Board and the Sierra Leone Ethics and Scientific Review Committee.

### 2.2. Data Collection Procedures

Peers and caregivers who consented and enrolled in this study completed a quantitative assessment battery that included measures on mental health and daily functioning. Trained study research assistants collected data from September 2019 through December 2019. The data collection timepoint for peers and caregivers overlapped with the post-intervention data collection timepoint for index participants (after all index participants had completed the YRI); thus, all data in this study on peers and caregivers were collected after a period of indirect exposure to the YRI.

### 2.3. The Youth Readiness Intervention (YRI)

The YRI is a culturally informed group intervention that includes core components of cognitive behavioral therapy, interpersonal therapy, and mindfulness techniques [5,33]. The YRI consists of 12 sessions that target development of emotion regulation skills, interpersonal skills, and problem-solving skills. Sessions typically incorporate a combination of psychoeducation, skill-building activities, and take-home practice. In the Youth FORWARD study, sessions were delivered 2–3 times per week by pairs of co-facilitators.

### 2.4. Measures

All quantitative measures were forward and backward translated from English to Sierra Leonean Krio in prior research, as per the WHO guidelines for translation and adaptation of measurement scales [34]. All measures have demonstrated strong internal consistency and cultural appropriateness. Peers and caregivers completed the same measures, with the exception of caregiver burden. We used the Hopkins Symptom Checklist (HSCL) a self-report symptom inventory designed to measure depression and anxiety symptoms (α = 0.92). It is commonly used in both clinical and research settings to assess mental health status and track changes in symptom severity over time [35]. Emotion regulation skills were evaluated with the Difficulties in Emotion Regulation Scale (DERS) (α = 0.65). The DERS is a self-report questionnaire used in both clinical and research contexts that assesses multiple aspects of emotion regulation, including emotional awareness, clarity, and acceptance, as well as impulse control and access to emotion regulation strategies [31]. Positive coping skills were assessed with the Brief COPE scale (α = 0.72–0.84), a self-report questionnaire used to assess a range of coping strategies used in response to stress, including adaptive (i.e., active coping, planning) and maladaptive (i.e., denial, substance use) approaches [36]. We focused on adaptative/positive coping skills. The Inventory of Socially Supportive Behaviors (ISSB) was used to assess social support (α = 0.87). The ISSB is a self-report measure that assesses the frequency of supportive behaviors received from others (i.e., emotional, informational, and practical support; [37]). Participant well-being was evaluated using the World Health Organization (WHO) Well-being-5 (α = 0.79; [38]), a short self-report scale assessing mental well-being through five positively worded items. It is widely used as a measure of emotional well-being in clinical and research settings due to its simplicity and reliability. The WHO Health-Related Quality of Life Inventory (BREF) (α = 0.85; [39,40,41]) and the WHO Disability Assessment Schedule (WHODAS) were used to assess functional impairment (α = 0.91; [34]). Caregivers also completed the Burden Assessment Scale (BAS; α = 0.89–0.91). The BAS is a self-report tool assessing the physical, emotional, social, and financial strain experienced by individuals providing care to others; it is commonly used in both clinical practice and research involving caregivers [42].

### 2.5. Data Analysis Approach

The network psychometrics framework guided data analysis [43,44]. In this framework, psychological constructs are represented as complex systems where each component—such as symptoms of a mental disorder or cognitive processes—can influence and be influenced by other components directly [24,25]. The core elements of network psychometrics include nodes and edges. Nodes represent observed variables, such as specific symptoms, behaviors, or a construct, such as anxiety. Edges symbolize the relationships between these nodes. This representation allows for the visualization and analysis of how each element within the network contributes to the broader construct (i.e., spillover effects or indirect benefits).

The overall topology of the network is a critical focus of analysis, which provides insights on properties such as connectivity, clustering, and path lengths to gain insights into the complexity and robustness of psychological phenomena. This type of analysis can reveal how tightly integrated or fragmented the network is, providing valuable information about the underlying structure of psychological constructs. A key aspect of network typology is the analysis of centrality measures. These measures include strength centrality, a measure of the importance or influence of a node within a network structure, and in a weighted network, refers to the sum of the weights of all edges attached to a particular node; betweenness centrality, which assesses how often a node appears on the shortest path between other nodes; and closeness centrality, which measures the average distance from a node to all other nodes in the network. These centrality measures help identify influential nodes (i.e., construct, symptom) within the network, which may be key targets for interventions or further study. Similarly, this approach also allows for the identification of clusters or groups of nodes that are more densely connected to each other than to the rest of the network. These clusters may represent meaningful substructures within the data, such as groups of symptoms that co-occur frequently or personality traits that tend to manifest together. Separate networks are produced for peers and caregivers and across treatment arms, and their structure and topology are analyzed and compared.

Considering the wide variety of data types used (continuous, count, and binary), we estimated Pairwise Markov Random Field networks. Specifically, Gaussian Mixed Graphical Models (GMGMs) were used. In GMGMs, mixed data types are accommodated by estimating the marginal and conditional relationships for different variable types (e.g., Gaussian for continuous data, logistic regression for binary data, Poisson for count data). These relationships are then encoded into a mixed covariance structure, which is subsequently inverted to derive partial correlations. Model selection was performed using the Extended Bayesian Information Criterion (EBIC), an extension of the Bayesian Information Criterion (BIC) that incorporates a penalty for the model’s complexity and is particularly suited for sparse networks, helping to avoid overfitting by discouraging the inclusion of unnecessary edges. The statistical significance of the edges was evaluated using an alpha level of 0.05. All the models were estimated using Bootstrapping (n = 5000) to assess the stability of the estimated network parameters, such as edge weights or node centrality estimates [45]. The topology of the networks was described based on local (strength, closeness, betweenness) and global network properties. All analyses were conducted using R statistical software (R Core Team, 2024, Vienna, Austria), and the Boonet package [45].

## 3. Results

### 3.1. Descriptive Statistics

Table 1 presents sample descriptive statistics. The mean age for peers was 30.99 years (SD = 9.27 for control and SD = 9.44 for YRI), with an age range of 16 to 64 years. Females comprised 44.7% of the total peer sample. The mean age was 37.50 years (SD = 12.90) for the control group and 37.75 years (SD = 2.51) for the YRI group, respectively. Females comprised 58.2% of the total caregiver sample. Regarding the personal network size of index participants in the analytical sample, 65.65% (n = 216) had a peer network size of three, 24.01% had a peer network size of two (n = 79), and 1.82% had a peer network size of one (n = 6).

### 3.2. Peer Models

#### 3.2.1. Peer Network Correlational Structures

**Figure 1:**
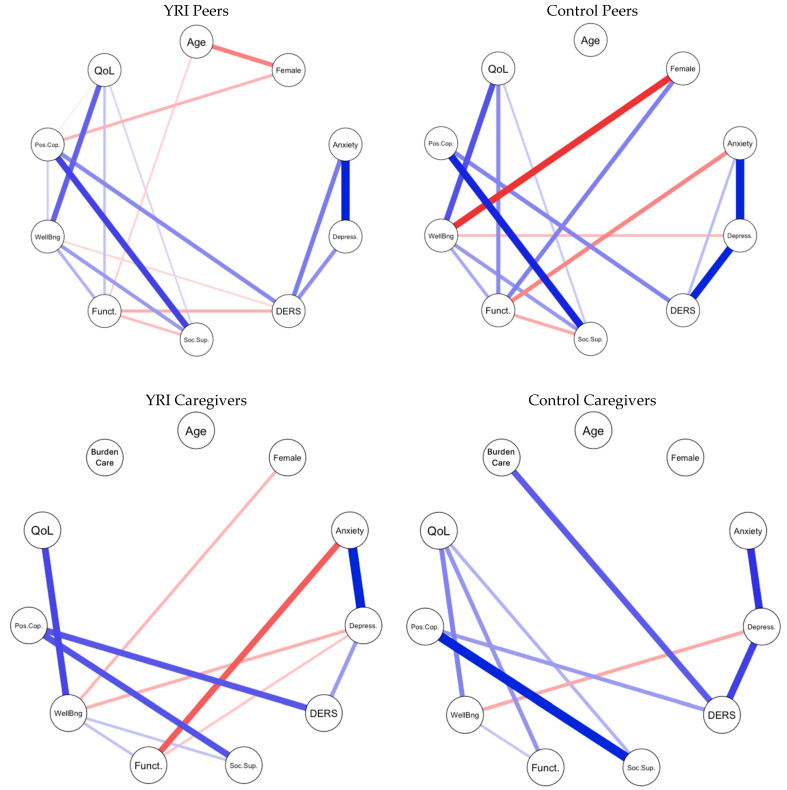
Network structures. Graph legend: Age = participants’ age; Depress. = depression; DERS = difficulties in emotion regulation; Soc.Sup = social support; Funct. = functioning; Wellbng = well-being; Pos.Cop. = positive coping skills; QoL = quality of life; BurdenCare = burden of care. Blue indicates a positive association between nodes; red indicates a negative association between nodes; bolder, thicker lines indicate a stronger association between nodes.

The peer network structures are displayed in Figure 1. In both networks (YRI and control), the results showed a positive association between emotion regulation and positive coping strategies, with a cluster of other mental health constructs strongly connected (anxiety, depression, and emotion regulation). Associations between anxiety and depression, depression and emotion regulation (stronger in the control network), and anxiety and emotion regulation (stronger in the YRI network) were particularly strong. However, the way in which this cluster of mental health symptoms was connected to the rest of the network differed across networks. In the control network, there was a significant negative association between anxiety and daily functioning, such that higher levels of anxiety symptoms were associated with lower daily functioning, controlling for the other associations in the network. Further, a significant negative association between depression and well-being was observed in the control network, in that elevated depression symptoms were associated with lower well-being. Regarding control participants’ demographics, female control peers showed lower well-being and higher functioning than male control peers.

When examining the YRI peers’ network structure, there was a negative association between poor emotion regulation and functioning, with worse emotion regulation related to worse functioning. In the YRI peer network, results showed weak but statistically significant associations between positive coping and well-being, as well as between positive coping and quality of life. Regarding demographic characteristics in the peer network, results showed weak but significant negative associations between age and functioning, as well as gender and positive coping. That is, as YRI peer network participants grow older, their daily functioning scores decrease, and females show fewer positive coping skills compared to males. The overall structure of associations between quality of life, well-being, functioning, social support, and positive coping skills was similar in both groups.

#### 3.2.2. Peer Network Centrality Measures

**Figure 2:**
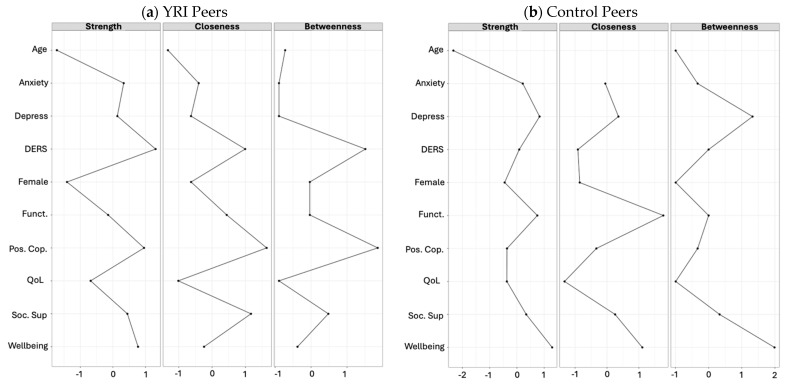

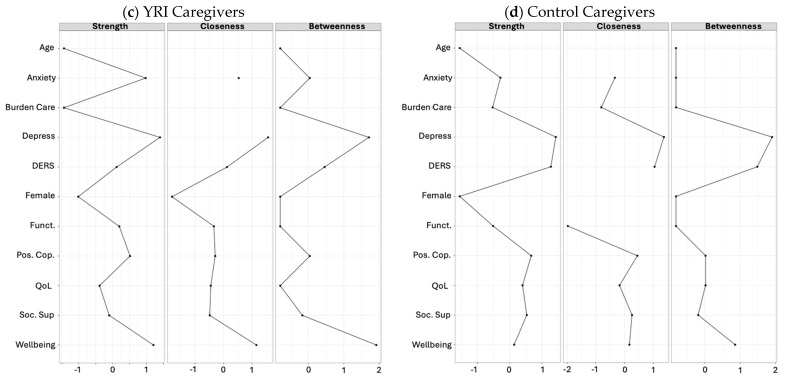
Network centrality measures (standardized estimates). Graph legend: Age = participants’ age; Depress. = depression; DERS = difficulties in emotion regulation; Soc.Sup = social support; Funct. = functioning; Wellbng = well-being; Pos.Cop. = positive coping skills; QoL = quality of life; BurdenCare = burden of care.

Peer network centrality measures are illustrated in Figure 2. When comparing the YRI and the control networks, the results of centrality measures suggest that well-being was a highly relevant construct in the peer control network. Well-being showed the highest strength centrality (larger association with all the other constructs) and betweenness centrality (common bridging role), and the second-highest closeness centrality (highest average proximity with other nodes). Functioning was also relevant, as it showed the second-highest strength centrality measure. Lastly, depression showed a high strength centrality and the second-highest betweenness centrality measure. Considering that the betweenness centrality measure shows how important a construct is in “bridging” other constructs in the network, this finding further supports the importance of depression “as a bridge” between the mental health cluster of variables and the rest of the network. In the YRI peer network, the most relevant construct was positive coping strategies, showing the highest betweenness and closeness centrality and the second-highest strength centrality. In the control peer network, depression and anxiety symptoms showed the lowest betweenness centrality.

### 3.3. Caregiver Models

#### 3.3.1. Caregiver Network Correlational Structure

Network structures for caregivers are displayed in Figure 1. Both the YRI and control networks significantly differed in their overall structure and patterns of associations. However, similar to the peer networks, in both the YRI and control caregiver networks, there was a cluster of mental health variables (anxiety, depression, emotion regulation) that were strongly connected to each other, with depression symptoms playing a bridging role for the constructs in the cluster.

In the control network, significant negative associations were found between well-being and depression (higher depression associated with lower well-being). Significant positive associations were found between emotion regulation and burden of care (worse emotion regulation related to a higher sense of caregiving burden), controlling for the associations between other variables. Significant positive associations between emotion regulation and positive coping were also observed. Lastly, the control caregiver network showed positive albeit weak associations between quality of life, well-being, functioning, and social support.

Regarding the YRI caregiver network, significant negative associations between gender and well-being were observed; female caregivers showed lower well-being scores than males when controlling for all other variables in the network. Functioning was negatively associated with anxiety and depression, and depression was negatively associated with well-being (higher depression associated with lower well-being), suggesting that the relationship between higher mental health symptoms, lower well-being, and lower functioning is stronger in the YRI caregiver network compared to the control caregiver network. Lastly, significant positive associations between emotion regulation and positive coping were observed (better emotion regulation associated with higher positive coping). In contrast to the control network, no significant associations were found between emotion regulation and sense of caregiving burden.

#### 3.3.2. Caregiver Network Centrality Measures

Centrality measures for caregivers are illustrated in Figure 2. Regarding centrality measures, the YRI and control caregiver networks do not substantially differ from the patterns found in the peer networks. For the caregiver networks, the most central construct was depression, displaying the highest strength and closeness centrality and the second-highest betweenness centrality. Emotion regulation was also relevant for both networks, particularly in the control caregiver network. In the YRI caregiver network, well-being was the second-most central construct, with the highest betweenness centrality.

## 4. Discussion

This study used a relatively novel analytic approach—network psychometrics—to explore patterns of association (or network structure) between mental health symptoms and risk/protective factors among peers and caregivers of youth who participated in an evidence-based mental health intervention in Sierra Leone. We also sought to understand ways in which these networks, or patterns of association, qualitatively differed between peers and caregivers of intervention participants and those of non-participants. To our knowledge, this is the first study to apply a network psychometric approach to understand how participation in an evidence-based mental health intervention (e.g., the YRI) might indirectly benefit close peers and caregivers of the intervention participants, and also the first study to apply this methodology in Sub-Saharan Africa. In partial support of our hypotheses, the findings suggest that the networks (or patterns of association) of mental health symptoms and risk/protective factors differed between YRI participants’ peers and caregivers and those of non-participants.

Results of models for peers highlighted several strong associations in the network structure of symptoms and risk/protective factors, as well as some differences in these patterns for peers of YRI participants and those of non-participants. For example, models reflected strong associations between depression and anxiety symptoms for both groups of peers, but peers of non-participants showed a stronger connection between higher depression symptoms and worse emotion regulation. Depressions symptoms and emotion regulation also showed high betweenness centrality in the network structure, indicating that these constructs may serve as a “bridging factor” in the network structure. This is in line with the extant literature supporting the association between poor emotion regulation and depression symptoms [46,47]. Given that the YRI specifically targets emotion regulation as a mechanism to improve mental health, indirect exposure to the YRI among peers may have helped improve their emotion regulation, which in turn might have mitigated depression symptoms. Alternatively, improvements in depression symptoms might have reduced the intensity or frequency of experiencing negative emotions (i.e., sadness, anger, fear), which could make regulating emotions less challenging [46,47]. In practical terms, identifying and targeting “nodes” with high betweenness centrality could help “disconnect” tightly linked symptoms or traits. Because the sample is cross-sectional, these possible explanations are speculative, and further research is needed to help establish directionality, causality, and/or mechanistic pathways. However, the findings point to connections between depression, anxiety, and poor emotion regulation among Sierra Leonean youth in the current study, as well as the potential for the YRI to indirectly benefit non-intervention-exposed peers via improvements in depression, anxiety, and emotion regulation.

Also of note, for peers of non-participants, being female was highly linked with lower well-being, but this association was not present for YRI participants’ peers. This difference might reflect the higher levels of daily hardships and environmental stressors that young females in Sierra Leone experience. For example, our prior research suggested similar patterns for females in that they were less educated than males (e.g., on average, males completed ~2 more years of education than females), reported higher levels of intimate partner violence, and cared for multiple children (e.g., average number of biological children in household = 3), all of which could impact experiences of well-being [22]. In contrast, being female was not associated with well-being among peers of YRI participants. Although the mechanism is unclear, indirect exposure to the YRI might help improve overall well-being in the contexts of hardship and adversity, including those associated with female identity status in Sierra Leone.

Further, models for both YRI participants’ and non-participants’ peers showed a strong association between higher social support and positive coping skills, and positive coping showed high centrality. Drawing from prior research [48], this association may support the importance of improving social support as a way to promote adaptive and healthy coping skills, or it might suggest that greater use of positive coping strategies can help bolster social connections [49,50]. This association could be more fully explored by using longitudinal study designs that incorporate mediation analysis in future research. Based on the current findings, targeting social support and positive coping could be important intervention targets for youth in Sierra Leone that could also “spill over” and benefit peers who have not been exposed to an evidence-based mental health intervention.

For caregivers, some associations in the network structures for YRI participants’ and non-participants’ caregivers mirrored those found in the peer network structures. For example, strong associations were also found between depression and anxiety symptoms for caregivers indirectly exposed and unexposed to the YRI, as well as between positive coping and social support. As with peers, these findings may guide a refined selection of intervention targets, both in terms of mental health symptoms as well as for protective factors, when tailoring evidence-based mental health interventions in resource-constrained settings.

Regarding patterns of association in the network structures that were unique to caregivers, findings for caregiving burden (which was not assessed for peers) differed between caregivers of YRI participants and those of non-participants. This is somewhat in line with prior findings in Sierra Leone exploring mental health spillover effects [12]. For caregivers of non-participants, a higher burden of care was strongly associated with worse emotion regulation, and worse emotion regulation was associated with higher levels of depression and anxiety. This points to the possibility of spillover effects. For caregivers of youth who did not participate in the YRI and thus likely continued to exhibit greater difficulties with mental health, interpersonal relationships, and daily functioning, the burden of care was likely greater than for caregivers of youth participants. For caregivers of YRI participants, the caregiving burden was not associated with mental health symptoms or risk/protective factors. This association may reflect the possibility of indirect benefits of the YRI that “spilled over” among caregivers, which is consistent with findings from our prior qualitative research [22]. Further research to understand the extent of these indirect benefits among household members could be relevant for policymaking decisions and increased financing for mental health interventions in Sierra Leone and other resource-constrained settings.

An additional difference in the network structures between YRI participants’ and non-participants’ caregivers worth noting relates to daily functioning, as assessed by the WHODAS. For YRI participants’ caregivers, findings showed a strong inverse association between daily functioning and anxiety symptoms. This pattern did not emerge for caregivers of non-participants. This may reflect potential indirect benefits of the YRI experienced among caregivers, considering that caregivers experienced reductions in perceived anxiety and distress (i.e., worry) following youth completion of the YRI, which may have helped improve their day-to-day functioning. More complex models in future research could potentially illuminate how and whether YRI participants’ mental health and functioning relate to and/or influence the patterns of association between mental health and risk/protective factors observed among caregivers.

## 5. Limitations

The study and analysis structure has several limitations to note. The findings of this study may not be generalizable, as it was completed in a rural part of Sierra Leone, where the sample had a lower educational level. Additionally, the YRI was implemented within an entrepreneurship program. While YRI participation has been associated with mental health benefits in prior studies (i.e., [5]), it is possible that the participants were unable to distinguish the YRI from the entrepreneurship training. Future studies might try to identify the unique contributions of different YRI components through factorial designs or conduction optimization trials, and then collect data from social networks (peers, caregivers) to better understand indirect benefits of specific YRI components. Lastly, these results assume social connectedness, and thus would not be applicable for individuals who are not socially connected with peers and/or caregivers.

While the network psychometrics analytical approach offers valuable insights into the complex relational structure of constructs, a few limitations should be acknowledged. First, although no extreme skewness exists across the variables used in the study, it is worth mentioning that Gaussian Mixed Graphical Models (GMGMs) may not fully capture the nuances of distributions with strong deviations from normality or heavily skewed count variables. Additionally, while the Extended Bayesian Information Criterion (EBIC) effectively promotes sparsity, its reliance on a pre-defined penalty parameter could lead to over- or under-selection of network edges if the parameter is not well calibrated. Bootstrapping provides robust stability metrics; however, it does not entirely eliminate potential biases due to sampling variability or small sample sizes in the original dataset, something particularly relevant to the caregiver sample in this study. Furthermore, the interpretation of local (e.g., centrality measures) and global network properties is inherently descriptive, and causal inferences cannot be reliably drawn from these analyses. Lastly, computational intensity, particularly with large datasets or high-dimensional networks, may limit the scalability and feasibility of this approach. These considerations underscore the importance of careful interpretation and transparency when applying this methodology to psychological data.

## 6. Conclusions

This study utilized a novel methodology and analysis approach to describe and compare the structure of mental health and daily functioning/well-being variables among peers and caregivers of youth participants in a behavioral intervention study. Our findings illustrating positive impacts within the peer and caregiver networks of YRI participants are promising, as they point to potential positive outcomes within the social networks of youth who receive mental health interventions. Future research should focus on this phenomenon using longitudinal designs that allow examination of mechanisms of such spillover effects (e.g., active communication about intervention activities and content, social learning through observing more positive behaviors). Research could also examine the use of family adjunctive tools (e.g., information sheets or workbooks that participants can share or work on with family members, additional family meetings during the intervention). It is also important for policymakers to be aware of the potential extended societal benefits of mental health care, as positive outcomes may diffuse within the social networks of youth who receive treatment. Overall, this initial examination of the diffusion of mental health benefits is promising and this area warrants further investigation.

## Figures and Tables

**Table 1:** Sample descriptive statistics.

	Peers			Caregivers		
	Control (N = 416)	YRI (N = 440)	Total (N = 856)	Control (N = 141)	YRI (N = 132)	Total (N = 273)
Age						
Mean (SD)	30.99 (9.27)	30.99 (9.44)	30.99 (9.36)	37.50 (12.90)	37.75 (12.51)	37.62 (12.69)
Range	16.30–64.00	16.50–63.90	16.30–64.00	17.70–80.00	18.70–64.30	17.70–80.00
Gender						
Female	171 (41.1%)	212 (48.2%)	383 (44.7%)	84 (59.6%)	75 (56.8%)	159 (58.2%)
Male	245 (58.9%)	228 (51.8%)	473 (55.3%)	57 (40.4%)	57 (43.2%)	114 (41.8%)
District						
Kono	106 (25.5%)	98 (22.3%)	204 (23.8%)	38 (27.0%)	32 (24.2%)	70 (25.6%)
Koinadugu	144 (34.6%)	164 (37.3%)	308 (36.0%)	42 (29.8%)	47 (35.6%)	89 (32.6%)
Kailahun	166 (39.9%)	178 (40.5%)	344 (40.2%)	61 (43.3%)	53 (40.2%)	114 (41.8%)

## Data Availability

The datasets used and analyzed in the current study can be made available upon reasonable request to the principal investigator.
